# Supplementary material for: Rapid turnover of life-cycle-related genes in the brown algae
Source: Genome Biol. 2019 Feb 14;20:35. doi: 10.1186/s13059-019-1630-6 (PMC6374913; doi:10.1186/s13059-019-1630-6)
Supplement: Supplementary file 3 — Protein alignments, corresponding Gblocks html file and CDS sequences for the lineage conserved gametophyte-biased genes. (ZIP 2109 kb) [file 13059_2019_1630_MOESM3_ESM.zip › Con_GA_alignments/Ec-18_000070.aa_ali.fasta-gb.htm]

Ec-18\_000070.aa.fasta.trans.aa\_ali.fasta


## Gblocks 0.91b Results

Processed file: **Ec-18\_000070.aa.fasta.trans.aa\_ali.fasta**  
Number of sequences: **2**  
Alignment assumed to be: **Protein**  
New number of positions: **449** (selected positions are underlined in blue)

```
                         10        20        30        40        50        60
                 =========+=========+=========+=========+=========+=========+
Ec-18_000070     MSEDAGRPLDPSRDRHREPADEKPRRRGSRPSGAAGGGSKKSTRRKSSSRAAAPAGAGGE
MSTRG.19305.1    ------------------------------------------------------------
                                                                             


                         70        80        90       100       110       120
                 =========+=========+=========+=========+=========+=========+
Ec-18_000070     RVREHGSDGPRRESGGLAASPDLALAQDKGSVRPAEAGSSRTRPGAKGVRRRIGGTDHDG
MSTRG.19305.1    ------------------------------------------------------------
                                                                             


                        130       140       150       160       170       180
                 =========+=========+=========+=========+=========+=========+
Ec-18_000070     GQVHAALGEERHQIAQRDQPVAERELGGSGGGVDRAILQKWRSDASAAVADDVGRNPALP
MSTRG.19305.1    ------------------------------------------------------------
                                                                             


                        190       200       210       220       230       240
                 =========+=========+=========+=========+=========+=========+
Ec-18_000070     DGRVAPQVPPDMEVEDEDCGGGYDDECFEEYSDEFDGEGEDEGPPPPVGKATAASVTRPG
MSTRG.19305.1    ------------------------------------------------------------
                                                                             


                        250       260       270       280       290       300
                 =========+=========+=========+=========+=========+=========+
Ec-18_000070     SARGEGGGAVVSPALKMRRPGDNIQAPDDKVLEELRDSLREENIAARERKVARPSPKPSE
MSTRG.19305.1    ------------------------------------------------------------
                                                                             


                        310       320       330       340       350       360
                 =========+=========+=========+=========+=========+=========+
Ec-18_000070     AATADVKSSSDAKAVRGAKGAKGSRSAAAAAATGGEASAAVAKGKFSVDASADVKRSRKG
MSTRG.19305.1    ------------------------------------------------------------
                                                                             


                        370       380       390       400       410       420
                 =========+=========+=========+=========+=========+=========+
Ec-18_000070     SGFKMADAKALTRALAKGDPRAERVRAIRGASRLEVESVRLFDQVPILPIDLYHRRLRGQ
MSTRG.19305.1    ---------------STGDPRTERVLAIRAACRLEVTSSRIFDQAPILPIDLYHRKLRGQ
                                  ###########################################


                        430       440       450       460       470       480
                 =========+=========+=========+=========+=========+=========+
Ec-18_000070     PALIRQAGTQTNDDARSVEGQTDEIVQSHKEVQFHLGHDDTALENLMRRLRSNRR-PPER
MSTRG.19305.1    PAIIRQAGSQTNEDARSVDVQTDEIFRVDKEAQFHLGHDDTALENLMRRLRSNRRGPAHL
                 #######################################################     


                        490       500       510       520       530       540
                 =========+=========+=========+=========+=========+=========+
Ec-18_000070     HQAKGEEGGQTQQRTVVDGGEDVPA--SGPGSATTTFKGIYNGNGMVAGTDPGEQEYPAP
MSTRG.19305.1    DHSPSAGMGESKGGDEGKGHQRTVAEEDGAVASTTTFKGLYNGR---KGVD--QDAFSAP
                                             ###############                 


                        550       560       570       580       590       600
                 =========+=========+=========+=========+=========+=========+
Ec-18_000070     RYGTDFD-GSS---GR---VSSAGGSDTLPPPLRLGKFLRQASVVMETLCEENLLHAPVK
MSTRG.19305.1    RYGKGVENGGSGGGGLIAGVGEAAALQPQPPPLRLGKFLRQASVVMETLCEENLLYASAA
                                    ######################################   


                        610       620       630       640       650       660
                 =========+=========+=========+=========+=========+=========+
Ec-18_000070     RRGGSHPLDPARGEHEDAVGRERGALFSGAGGREGEGWEEVGAEGGVFGAGARGATAAAT
MSTRG.19305.1    SSSPLEAAAGMRGEEEAHDDRQGRSLFSERASRGAEGWEEVGGAG----SWLLGPGVAAP
                            ##################################        #######


                        670       680       690       700       710       720
                 =========+=========+=========+=========+=========+=========+
Ec-18_000070     EDTGSSTAEEKR--RQGGDEGRVRGR-GAGALLEGSEVVGVEFSKVKRSMLVTAHARPVG
MSTRG.19305.1    VDTGRPVEEEKRPEADGVSVGNCDTRVGPGRLLEGSKVVGIEFSRVKRSMLVTAHARPAS
                 ############    ########## ###############################  


                        730       740       750       760       770       780
                 =========+=========+=========+=========+=========+=========+
Ec-18_000070     R---RRRSEDGGGGGG-GGGTDEREAVLEGCGVVCVWNTENVQGAPVSVLCGEGLFSCLC
MSTRG.19305.1    QSWKRRRSADGSGGGGDSSNIPEYEAILEGCGVICVWNADSPKAAPVSVLCGEGLFSCLC
                     ############      ######################################


                        790       800       810       820       830       840
                 =========+=========+=========+=========+=========+=========+
Ec-18_000070     LARHQAHVVITGTEEGCLLLWDLRETASEGRAAESRELGLHCGIRAPTYVTAGGDGRGED
MSTRG.19305.1    LPLHHAHTVVAGTAEGCVLLWDLREPASSSRAAESRSLRLRCGIRSPTYSTAGGGG--GD
                 ########################################################   #


                        850       860       870       880       890       900
                 =========+=========+=========+=========+=========+=========+
Ec-18_000070     GGLSKAGCGHSSKVVSVVAIPSPTGGVGMDGGGGGIG-GMISLGDACSFQVASLDDRGTV
MSTRG.19305.1    AGTSGPGGGHVSTVVSVIPIPVAAGG-GMSGNGGFSGEGFISLEEANSFQIASLDDRGTV
                 ########################## ########## ######################


                        910       920       930       940       950       960
                 =========+=========+=========+=========+=========+=========+
Ec-18_000070     SIWLASEVPRGDEGGSQTDLGLSPGGRVRLALSKALRPGDQPSLAAAVRPRGGGGAVPVS
MSTRG.19305.1    SIWLASEVSRADEGGSQSDLGLSPGGRIRLALSKSLHPG------AQV------------
                 #######################################                     


                        970       980       990      1000      1010      1020
                 =========+=========+=========+=========+=========+=========+
Ec-18_000070     VPRFSTSTRNRLDQTTDTTASSAAAGSARALAFSPEDPNRFLIGDSSDRVTHASRLGKPP
MSTRG.19305.1    --------------------------------------------------QH--------
                                                                             


                       1030      1040      1050      1060      1070      1080
                 =========+=========+=========+=========+=========+=========+
Ec-18_000070     PPRAYGPPRRPGERRLDGGGGGTGEEREGAMGGVTCLAFSPFFPKYFLAGCGDGSVRLYN
MSTRG.19305.1    ------------------------------------------------------------
                                                                             


                       1090      1100      1110      1120      1130      1140
                 =========+=========+=========+=========+=========+=========+
Ec-18_000070     EASVVPLTTWEAGSTAAADSRRNGGSSSSWPAAISSLAWSEHRPAVFFALDVLGMVHAFD
MSTRG.19305.1    ------------------------------------------------------------
                                                                             


                       1150      1160      1170      1180      1190      1200
                 =========+=========+=========+=========+=========+=========+
Ec-18_000070     LLEDDAGPVASEGCPDASVGVMERSRSPTNPPAAVVEDASSSSLALPTLALSNETLATGR
MSTRG.19305.1    ------------------------------------------------------------
                                                                             


                       1210      1220      1230      1240      1250      1260
                 =========+=========+=========+=========+=========+=========+
Ec-18_000070     RPKVALAFGGSVFAKTLAGRMFRQSRSPPVADGVERSEAGVPEVSERERMKEWLGEVLWA
MSTRG.19305.1    -----TTLGGSVV-----------------------------------------------
```

```
Parameters used
Minimum Number Of Sequences For A Conserved Position: 2
Minimum Number Of Sequences For A Flanking Position: 2
Maximum Number Of Contiguous Nonconserved Positions: 8
Minimum Length Of A Block: 10
Allowed Gap Positions: None
Use Similarity Matrices: Yes
```

```
Flank positions of the 12 selected block(s)
Flanks: [378  475]  [509  523]  [560  597]  [612  645]  [654  672]  [677  686]  [688  718]  [725  736]  [743  836]  [840  866]  [868  877]  [879  939]  

New number of positions in Ec-18_000070.aa.fasta.trans.aa_ali.fasta-gb:  449  (35% of the original 1260 positions)
```
